# Post-myocardial Infarction Ventricular Septal Rupture Complicated by Cardiogenic Shock Stage D: A Successful Case of Extracorporeal Membrane Oxygenation as a Bridge to Delayed Surgical Repair

**DOI:** 10.7759/cureus.50574

**Published:** 2023-12-15

**Authors:** Maria Rojas-Espinoza, Celia Aguilar-Mejía, Juan Manuel Muñoz-Moreno

**Affiliations:** 1 Perioperative Cardiology, Instituto Nacional Cardiovascular, Lima, PER; 2 Cardiology, Instituto Nacional Cardiovascular, Lima, PER

**Keywords:** ventricular septal rupture, myocardial infarction, extracorporeal membrane oxygenation, cardiogenic shock, cardiac surgical procedures

## Abstract

Ventricular septal rupture (VSR) after myocardial infarction is often complicated by cardiogenic shock (CS) with high in-hospital mortality rates. Early use of preoperative venoarterial extracorporeal membrane oxygenation (VA ECMO) and delayed surgical repair have demonstrated lower mortality rates; however, the optimal timing of surgical intervention remains controversial. We report the case of a 53-year-old man with CS stage D due to post-myocardial infarction VSR, who was successfully treated with VA ECMO as a bridge to delayed surgical repair. This case highlights the complexity of determining the optimal timing for surgical intervention in these patients and emphasizes the benefits of early use of VA ECMO for preoperative stabilization in patients with CS and multiorgan failure.

## Introduction

Ventricular septal rupture (VSR) is an uncommon mechanical complication of myocardial infarction that occurs between the third and fifth day of evolution and is usually complicated by cardiogenic shock (CS) resulting in high in-hospital mortality [1,2]. The optimal timing of intervention remains controversial [1], especially in patients with CS and multiorgan failure, where the early use of mechanical circulatory support (MCS) devices such as peripheral venoarterial extracorporeal membrane oxygenation (VA ECMO) in the preoperative period and delayed surgical repair of VSR have been associated with lower mortality [3,4]. This improved outcome with delayed surgery may be related to better myocardial tissue stability allowing for more effective repair [4].

We present the case of a 53-year-old man with CS stage D secondary to post-myocardial infarction VSR, in whom VA ECMO was used as a bridge to successful delayed surgical repair.

## Case presentation

A 53-year-old man was admitted to our hospital with oppressive chest pain associated with dyspnea for three days. His medical history was significant for hypertension and heavy smoking. Physical examination revealed a left parasternal holosystolic murmur and crackles in the lower third of both lungs. Blood pressure was 102/75 mmHg, pulse was 130 beats/minute, respiratory rate was 26 breaths/minute, and oxygen saturation was 96% with an FiO_2_ of 0.36. The electrocardiogram showed sinus rhythm and ST-segment elevation in precordial leads. The troponin level was elevated (5.2 ng/mL; normal <0.1 ng/mL). The diagnosis of anterior ST-elevation myocardial infarction complicated by VSR was raised.

**Figure 1:**
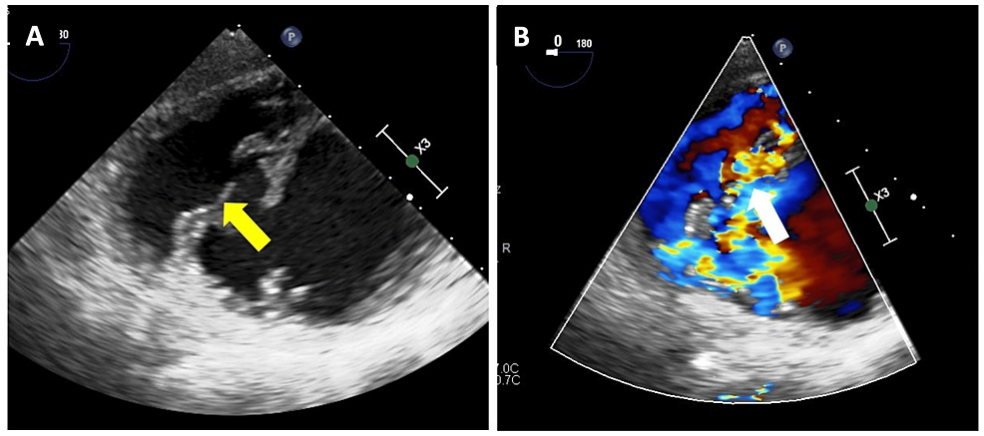
Transesophageal echocardiography. (A) Large and complex ventricular septal rupture (VSR) of 17 mm (yellow arrow). (B) Color Doppler in the area of the VSR with a left-to-right shunt (white arrow).

Transesophageal echocardiogram (TEE) showed a left ventricular ejection fraction (LVEF) of 47%, right ventricular fractional area change (RVFAC) of 33%, and the presence of an apical VSR of 17 mm along with left-to-right shunting (Figure 1).

**Figure 2:**
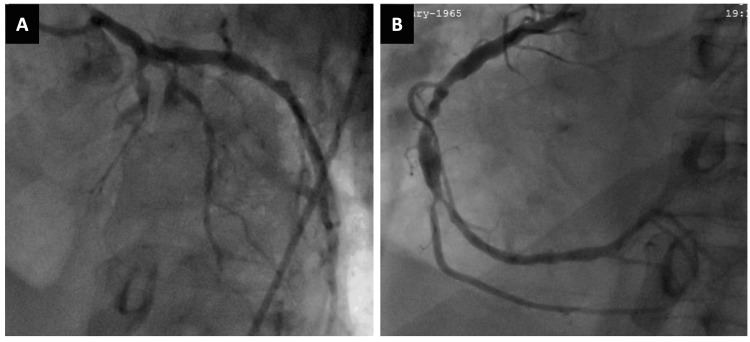
Coronary angiogram. (A) The left anterior descending artery occluded in the middle third. (B) The right coronary artery with severe stenosis in the middle third.

Coronary angiography revealed occlusion of the mid-left anterior descending (LAD) artery and severe stenosis of the mid-right coronary artery (Figure 2).

Right heart catheterization revealed a Qp/Qs ratio of 2.68, cardiac index of 1.25 L/minute/m^2^, pulmonary capillary wedge pressure of 39 mmHg, and right atrial pressure of 21 mmHg. His clinical condition deteriorated and was complicated by CS stage C, for which he was intubated and connected to mechanical ventilation, started on norepinephrine 0.5 µg/kg/minute, dobutamine 7.5 µg/kg/minute, and intra-aortic balloon pump (IABP) was implanted. On day two of hospitalization, renal and hepatic deterioration and lactate elevation (4.2 mmol/L) were added, progressing to CS stage D, for which it was decided to implant emergency peripheral VA ECMO guided by TEE.

Clinical evolution after placement of VA ECMO was favorable, and 12 days after myocardial infarction, surgical repair with bovine pericardial patch of the VSR was performed, as well as placement of three coronary artery bypass grafts (left internal mammary artery to LAD, saphenous vein to diagonal, and saphenous vein to posterior descending). In the immediate postoperative period, we continued to wean VA ECMO and IABP with LVEF of 40% and RVFAC of 35% and maintained dobutamine support at 5 µg/kg/minute, which was gradually tapered.

**Figure 3:**
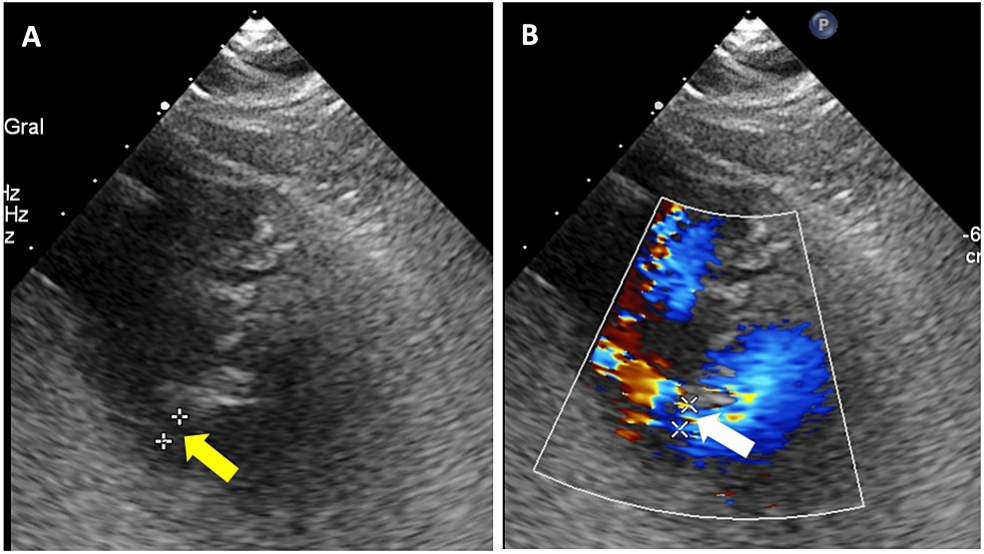
Pre-discharge transthoracic echocardiography. (A) Parasternal short-axis view showing a residual ventricular septal rupture (VSR) of 3 mm (yellow arrow). (B) Color Doppler in the area of the residual VSR (white arrow).

The pre-discharge transthoracic echocardiogram showed an LVEF of 40% and a residual interventricular defect of 3 mm adjacent to the pericardial patch, which did not cause significant hemodynamic compromise (Figure 3).

The evolution was favorable, and he was discharged one month after hospitalization on aspirin 100 mg od, clopidogrel 75 mg od, atorvastatin 40 mg od, valsartan 80 mg bid, bisoprolol 5 mg od, dapagliflozin 10 mg od, spironolactone 50 mg od, and furosemide 40 mg od.

At six months of outpatient follow-up, the patient remains in functional class II, continues to receive optimal medical therapy, and has had no new ischemic episodes or rehospitalizations.

## Discussion

The ideal timing of surgical intervention for post-myocardial infarction VSR complicated by CS remains controversial, as higher mortality has been reported when surgery is performed acutely compared to delayed intervention [1]. It has been reported that surgery in the first 24 hours has the highest mortality (>60%), within the first seven days the mortality is 54.1%, compared to after seven days when the mortality decreases to 18.4% [5,6]. In our country, a mortality of 50% has been reported in patients with isolated VSR, and CS has been identified as one of the main complications (41.7%) in them [2].

Patients with post-myocardial infarction VSR with CS and multiorgan failure usually present with a large VSR or an infarct with biventricular involvement [1], requiring more efficient MCS, such as VA ECMO, to achieve hemodynamic stability, improve preoperative status, and allow delayed surgery [1,4].

The time elapsed between myocardial infarction and surgical repair of VSR has an impact on patient survival [7]. Ariza et al. performed a retrospective study (from 2014 to 2017) among 28 patients with post-myocardial infarction VSR complicated by CS, and found that only the group of patients who underwent early MCS with VA ECMO as a bridge to delayed surgery (17.9%) survived to hospital discharge compared to those who underwent unsupported surgery, postoperative MCS, and conservative management, whose mortality was 27.3%, 50%, and 100%, respectively [3]. Delayed surgery in patients using early VA ECMO had a mean of 5.2 days (range = 4-6 days) after admission [3]. Arnaoutakis et al. reported that the longer the interval between myocardial infarction and surgical repair of VSR, the better the outcomes, especially when surgery was performed after seven days, highlighting that mortality after day 21 was reduced by up to 10% [6,7]. In our case, VA ECMO was used as a bridge to delayed surgery (12 days after infarction), which was successful after stabilizing and improving the patient’s hemodynamics and organ function.

The complete maturation of the VSR edges provides a more durable and resistant tissue for the placement of sutures to secure the patch [7], which may explain the better results with delayed surgery. In addition, it is important to highlight the benefit of VA ECMO in reversing multiorgan failure. However, it is worth mentioning that the use of this type of MCS device is also associated with in-hospital complications such as bleeding and infection [3], which is why it should be used for the minimum time necessary. The mean duration of early VA ECMO support in the group of patients who survived to hospital discharge was nine days (range = 4-12 days) [3]. Our patient spent a total of 10 days on ECMO-VA, and in the immediate postoperative period, weaning was successful without complications.

## Conclusions

The reasonable use of VA ECMO as a bridge to delayed surgery for post-myocardial infarction VSR complicated by CS has shown benefits in survival and postoperative outcome; however, the optimal timing of surgery remains controversial, reflecting the complexity of these cases.

Our report highlights the usefulness of early support with VA ECMO to improve hemodynamic stability and organ function in the preoperative period and supports the trend of delayed surgery in this group of patients.
